# Assessment of Diarrhea and Its Associated Factors in Under-Five Children among Open Defecation and Open Defecation-Free Rural Settings of Dangla District, Northwest Ethiopia

**DOI:** 10.1155/2018/4271915

**Published:** 2018-09-12

**Authors:** Abireham Misganaw Ayalew, Worku Tefera Mekonnen, Samson Wakuma Abaya, Zeleke Abebaw Mekonnen

**Affiliations:** ^1^Federal Ministry of Health, Addis Ababa, Ethiopia; ^2^Addis Ababa University, College of Health Sciences, Addis Ababa, Ethiopia

## Abstract

**Background:**

Open defecation (OD) is a widespread problem in the developing world. This practice facilitates the transmission of diarrheal diseases. In Ethiopia, still the national open defecation rate in 2014 was 34.1% (37.9% in rural and 8.7% in urban).

**Objective:**

To assess diarrheal morbidity in under-five children and its associated factors in Dangla district, Northwest Ethiopia, 2016.

**Methods:**

A community-based comparative cross-sectional study design with a multistage random sampling technique was applied. The total sample size was 550 (275 ODF and 275 OD). Descriptive and inferential statistics were done.

**Results:**

A total of 525 participants were interviewed making the response rate 95.45%. The prevalence of diarrhea was 9.9% in ODF and 36.1% in OD kebeles. In ODF kebeles, child immunization (AOR = 0.037; 95% CI: 0.006–0.243), latrine presence (AOR = 0.036; 0.006–0.233), water shortage (AOR = 8.756; 95% CI: 1.130–67.831), and solid waste disposal (AOR = 0.143; 95% CI: 0.020–0.998) have statistically significant association with diarrhea occurrence. While in OD kebeles child immunization (AOR = 0.032; 95 CI: 0.008–0.123), water access of 7.5–15 liters/day (AOR = 0.029; 95% CI: 0.006–0.152), water shortage (AOR = 18.478; 95% CI: 4.692–72.760), and proper solid waste disposal (AOR = 0.023; 95% CI: 0.005–0.117) have significant association with diarrhea occurrence.

**Conclusions:**

The overall prevalence of under-five diarrhea was low in ODF kebeles as compared with OD kebeles. The study showed that child immunization, latrine presence, water shortage in household, and solid waste disposal practices had statistically significant association with diarrhea occurrence in ODF kebeles, while water access at the individual level, water shortage in household, child immunization, and solid waste disposal have statistically significant association with diarrhea occurrence in OD kebeles. Integrated efforts are needed from the Ministry of Health together with line ministries and developmental partners in improving latrine utilization at household level, water shortage in households, and solid waste disposal practices.

## 1. Introduction

The majority of OD practices, referred to in national surveys as defecating in fields, forests, bushes, bodies of water, or other open spaces, take place in rural areas of low-income countries [1]. Worldwide diarrheal disease is the second leading cause of death in under-five children responsible for 1.7 million morbidity and 760, 000 mortality every year [2]. In Ethiopia, diarrhea kills half million of under-five children annually next to pneumonia [3].

According to 2014 Joint Monitoring Program (JMP), OD was reducing by half in developing regions from 1990 to 2012. About 825 million people practicing OD reside in just 10 countries of which five are in Africa (Nigeria, 39 million; Ethiopia, 34 million; Sudan, 17 million; Niger, 13 million; and Mozambique, 10 million) [4]. In the rest of the world, the number of people practicing OD is estimated to be 182 million [1, 4].

In sub-Saharan Africa, it is estimated that 215 million people that is 8% of the urban population and 35% of the rural population continue to engage in OD [1]. In Ethiopia, still the national OD rate in 2014 was 34.1% (37.9% in rural and 8.7% in urban) [5]. This practice facilitates the transmission of diarrheal diseases, one of the leading causes of mortality in under-five children in sub-Saharan Africa [6].

Total sanitation approaches, the most promising approaches aimed at empowering communities as a whole to become “OD FREE,R aimed at raising awareness of the risks associated with OD and generating a collective sense of intolerance towards OD [7].Nevertheless, it has been suggested that total sanitation approaches can result in rapid significant improvements and hold promise for decreasing OD in sub-Saharan Africa [8]. In Ethiopia, the Southern Nations, Nationalities, and Peoples' Region (SNNPR) adapted CLTS to local Ethiopian conditions in the 2003 Gregorian calendar and achieved a remarkably rapid reduction in OD. The approach was scaled up and mainstreamed in the National Sanitation Strategy and integrated into the Health Extension Worker program. Poor sanitation, lack of access to clean water supply, and inadequate personal hygiene are responsible for 90% of diarrheal disease occurrence; these can be easily improved by health promotion and education [3].

It is estimated that 1.7 billion cases of diarrhea occur every year, causing 800,000 deaths among children under 5 years of age worldwide [9], and 15% of the global population still engage in OD [1]. It also underlines that OD leads to deadly diarrhea and other intestinal diseases which kill hundreds of thousands of children every year. Evidences also showed that about 80% of the rural and 20% of urban sub-Saharan population have no access to safe water and sanitation [10].

In Ethiopia, three-fourths of the health problems of under-five children are communicable diseases which come from the environment, specially water and sanitation [11]. Diarrhea is the leading cause of under-5 mortality causing 23% of deaths and around 44% stunted [4]. In Ethiopia, over 75–80% of the communicable diseases are caused due to poor environmental health conditions arising from unsafe and inadequate water supply and poor hygienic and sanitation practices [11].

An estimated 64,540 children could be saved every year by improving water, sanitation, and hygiene in the country [4]. It is critical to understand what factors influence the pace to reduce OD in order to develop effective strategies to improving sanitation and reducing diarrhea morbidity and mortality caused by the lack of sanitation. Furthermore, there is a need for more realistic targets for global campaigns that will put forth following the MDGs that ended in 2015.

To date, information on the impact of being the ODF kebele on under-five diarrhea is limited or meager as a comprehensive package. It is hypothesized that people living in the ODF kebeles will have reduced diarrheal-related morbidity and mortality compared with people living in OD kebeles. However, all exposure routes to the increased burden of disease must be considered.

The knowledge of existing gaps in the differences between ODF and OD kebeles can be linked to a proper problem inventory. This research also helps to explore the basics of the ODF status in the prevention of diarrhea and also what changes can come after the ODF verification which in turn plays a major role in the proper planning and monitoring of sanitation and hygiene activities and programs that contribute for diarrhea prevention.

## 2. Materials and Methods

**Figure 1:**
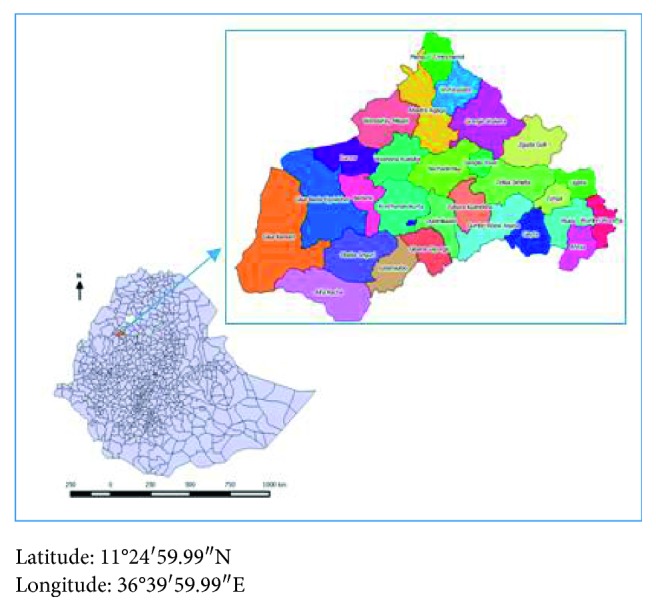
Map of the study area.

*Study Area*. The study was conducted in Dangla woreda, Awi zone, a city located 480 kilometers away from the capital city Addis Ababa. In this woreda, there are 27 rural kebeles (kebeles are smaller administrative units); 15 of the kebele were ODF, and 19309 under-five children are living in the woreda. The map shows the kebele under the Dangla district (Figure 1).

*Study Design and Period*. The study was a community-based comparative cross-sectional study conducted from July 2015 to June 2016.

*Source Population*. All children under five years of age in the Dangla district.

*Study Population*. Caregivers and children under five years of age living in ODF and OD settings of Dangla woreda.

*Inclusion Criteria*. Those households that have at least one child under five years of age in the house residing in the ODF or OD kebeles.

*Exclusion Criteria*. Those households located in the urban settings.

### 2.1. Sample Size Calculation

The sample size was calculated by Epi Info version 7 software (source: CDC) using double-proportion formula with the following assumptions:

- From a study done in Sidama Zone [12], diarrhea prevalence in ODF kebele (P1) = 24.7% and in OD kebele (P2) = 26.5%
- 95% confidence interval
- 80% power of test
- Ratio between ODF and OD kebeles =1
- To detect odds ratio of =2

Having the assumptions above, the sample size was 332. Since multistage sampling was used for this particular study, the design effect of 1.5 was considered and then the sample size became 498. With 10% contingency, the total sample size was determined to be 550 (275 for non-ODF and 275 for OD free).

### 2.2. Sampling and Data Collection Techniques

A multistage sampling technique was applied, the kebele from each group was selected randomly by the lottery method, and the samples were distributed proportionally by their number of under-five children. Children under five years of age residing in ODF and OD households were identified through a house-to-house enumeration prior to the actual data collection using the family folder by health extension workers. For a household having two or more children, we have taken the youngest child. The actual data collection was carried out in February 2016 by trained nursing students using a structured questionnaire. An observation checklist was also applied to assess latrine utilization.

### 2.3. Data Collection Instruments

A structured questionnaire which was adopted from different literatures was used, and an observation checklist was also applied to assess latrine utilization.

### 2.4. Variables

Dependent variable is the occurrence of diarrheal diseases in children under five years of age. Independent variables are as follows:

- Household income
- Sociocultural factor
- Hygiene and sanitation variables
- Household water access, treatment, and storage
- Hygiene and sanitation perception and practices
- Maternal and child health factors

### 2.5. Operational Definitions

#### 2.5.1. ODF

A status given by the woreda by their latrine, hand washing, household water access, and safe storage coverage and practices as per the ministry of health ODF verification and certification criteria.

#### 2.5.2. Diarrhea

A disease characterized by frequent (>3) loose or watery stools in 24 hours, or a single stool with blood/mucus with a 14-day prior to study.

#### 2.5.3. Unimproved Water Sources

Unprotected dug well, unprotected spring, cart with small tank or drum, surface water (e.g., river, dam, lake, pond, stream, canal, or irrigation channel), and bottled water.

#### 2.5.4. Proper Disposal

A way of disposal refuses that which included burning, burying in a pit or storing in a container, and disposing in the designed site.

### 2.6. Data Quality Control Measures

During data collection, kebeles were coded, all field workers were trained prior to data collection, and regular supervision was done during the field work. Each data collector checked the questionnaires for completeness before leaving each study participant. All filled questionnaires were reviewed at the end of the day by the supervisor. Original questionnaire was prepared in English and then translated into Amharic and translated back to English to check consistency, and the pretest was done on the data collection instrument before conducting the study.

### 2.7. Data Analysis

Data were coded and entered into Epi Info version 7 and cleaned and checked for completeness. Initially, descriptive statistics were done to determine the diarrhea prevalence and to characterize the variables' frequency and percentages. Bivariate and multivariate analyses were done using SPSS version 20 software. In the multivariate analyses, an Enter method was used and a statistical significance of 0.05 was considered. Results were presented using tables, graphs, and charts.

## 3. Results

### 3.1. Descriptive Results

A total of 525 (263 households from ODF and 262 households from OD) participants were interviewed making the response rate 95.45%. Among the respondents, 162 (61.8%) from ODF kebeles and 112 (42.6%) from OD kebeles were females. The total respondents' age ranged from 17–75 years with a mean (SD) age of 39.14 (±11.97) (Table 1).

As shown in Table 2, the prevalence of diarrhea among ODF kebeles was 9.9%, whereas in OD kebeles, it was 36.1%. From the total diarrhea cases, the majority 121 (77.9%) of under-five diarrhea cases were from those OD kebeles, but the magnitude of under-five diarrhea in ODF kebeles was 26 (22.1%).

The study also revealed that majority of respondents in both ODF (235 (89.7%)) and OD (244 (92.8%)) kebeles have a private latrine. From those households that have latrines, majority (87.4%) of respondents from ODF kebeles and 87.8% of respondents from OD kebeles have traditional pit latrines, respectively (Table 3).

Regarding waste disposal, 90 (34.4%) of households from ODF and 156 (59.3%) of households from OD kebeles dispose openly their solid waste, while 20 (7.6%) from ODF and 117 (44.5%) from OD kebeles dispose their liquid waste openly (Table 4).

**Figure 2:**
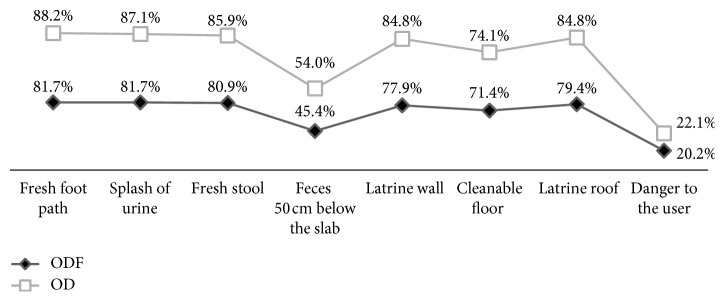
Observation result of households that have a latrine in Dangla woreda, Northwest Ethiopia, 2016.

From the observations made in latrines, a majority of 88% in OD and 82% in ODF kebeles had fresh foot path and splash of urine. Similarly, 86% of latrines in OD kebeles and 81% in ODF kebeles had fresh stool during observations (Figure 2).

### 3.2. Multivariate Analysis Result in ODF Kebeles

The multivariate analysis result showed that child immunization, latrine presence, water shortage in the household, and solid waste disposal had a statistically significant association with diarrhea occurrence in ODF kebeles.

Keeping other factors constant, immunized children were 96% less likely (AOR = 0.037; 95% CI: 0.006–0.243) to have diarrhea as compared to nonimmunized children, and the association was statistically significant. Similarly, those households having a latrine were 96.4% less likely (AOR = 0.036; 95% CI: 0.006–0.233) to have diarrhea as compared to those who did not have a latrine, and the association was statistically significant.

Regarding water shortage, holding other factors constant, households with water shortage had 8.7 times higher chance (AOR = 8.756; 95% CI: 1.130–67.831) of diarrhea occurrence as compared to the reference category. Pertaining solid waste disposal, those who dispose safely were 86% less likely (AOR = 0.143; 95% CI: 0.020–0.998) to have diarrhea as compared to those who dispose openly, and the association was statistically significant (Table 5).

### 3.3. Multivariate Analysis Result in OD Kebeles

The multivariate analysis result showed that child immunization, water access at the individual level, water shortage in the household, and solid waste disposal had statistically significant association with diarrhea occurrence in OD kebeles. Keeping other factors constant, immunized children were 97% less likely (AOR = 0.032; 95 CI: 0.008–0.123) to have diarrhea as compared to nonimmunized children, and the association was statistically significant. Regarding water access at the individual level, water access of 7.5–15 liters/day reduces the chance of diarrhea occurrence by 97% (AOR = 0.029; 95% CI: 0.006–0.152) and water access of greater than 15 liters/day reduces the chance of diarrhea occurrence by 93% (AOR = 0.068; 95% CI: 0.010–0.474) as compared to the reference category, and the association was statistically significant.

Pertaining to water shortage, households with water shortage had 18 times higher chance (AOR = 18.478; 95% CI: 4.692–72.760) of diarrhea occurrence as compared to households having no water shortage. Keeping other factors constant, safe waste disposal reduces the chance of diarrhea occurrence by 97% (AOR = 0.023; 95% CI: 0.005–0.117) as compared to open disposal, and the association was statistically significant (Table 5).

## 4. Discussion

To date, scientific evidence on the impact of the ODF kebele on under-five diarrhea and the factors that play a role in its implementation are meager as a comprehensive package. Hence, this research explored the basics of the ODF status in the prevention of diarrhea that contributes for designing effective interventions at different segments of the health system.

Accordingly, the prevalence of diarrhea among ODF kebeles was found to be 9.9%, while in OD kebeles, it was 36.1% showing significant differences. The prevalence in ODF kebeles was still much lower as compared with the study done in ODF kebeles of Sidama zone, and in SNNPR, the prevalence was 24.7% [12]. The reason behind the decrement of diarrhea prevalence in the ODF study area may be due to the quality of ODF declaration, certification, and follow-up. After ODF declaration, this follow-up may also be related with the commitment of the region, zone, woreda, and kebele's ODF verification and certification teams.

The prevalence of under-five diarrhea in OD kebele (36.1%) was higher as compared with the study done in Sidama zone which was 26.5% [12], and in a study in India, the prevalence was 15.9% in OD kebeles [2], but it is almost similar with national under-five diarrhea prevalence that ranges from 11% to 38% [11]. Differences might be because of the people's way of life, educational levels of the community, maternity care of child's immunization, and nutritional differences that are not related with the status of being ODF or OD.

The prevalence of under-five diarrhea among ODF and OD kebeles had a statistically significant difference with *X*^2^ = 50.791 and *P*=0.001, but the study done in Sidama zone showed that there is no significant difference between the two communities with *X*^2^ = 0.721 and *P*=0.396. The difference with a study in Sidama might come because of the differences in the quality of the ODF status.

Latrine presence has showed a statistically significant association with diarrhea occurrence both in ODF and OD kebeles in which the odds of developing diarrhea for families having a latrine was 96.4% times lower in ODF kebeles and 61.8% times lower in OD kebeles. The percentage of the latrine in ODF kebeles that is less than OD kebeles indicates that individuals living here are still practicing OD, and in the true sense, these are not ODF kebeles. The result is consistent with a study in Mali [13]. This is also in line with ODF verification and certification guideline and CLTSH implementation guideline [14, 15]. The study from the Derashe district of SNNPR also supports this finding in which the odds of developing diarrhea was 2.43 times higher among children of families who had no latrine when compared to children of families who had latrine [16].

In ODF kebeles, immunized children were 96% less likely to have diarrhea as compared to nonimmunized children which is much comparable with the result from OD kebeles that showed the odds of immunized children were 96.8% less likely to have diarrhea as compared to nonimmunized children. The similarities between these two settings come because of the socioeconomic, agroecological, MCH services, and way of life similarities since they are administered in one district. This finding is inconsistent with a result from Eastern Ethiopia where immunization had no significant association with diarrhea occurrence in under-five children [17].

Regarding water shortage in OD kebeles, households with water shortage had 18 times higher chance of diarrhea occurrence as compared to households having no water shortage which is much higher as compared with the result in ODF kebeles where water shortage increases the chance of diarrhea occurrence by eight times. This could be explained by the fact that households having water shortage could not keep their personal hygiene as necessary. Results from both settings are not comparable with a result from the Derashe district of SNNPR in which diarrhea among children have no statistically significant association with water shortage [16].

The analysis result from OD kebeles also showed that those households who got minimum standard (7.5–15 liters/day) water access at the individual level reduce the chance of diarrhea occurrence by 97%. This result is linked with the recommendation from the sphere handbook of water, sanitation, and hygiene for humanitarian charter and minimum standard that average water use for drinking, cooking, and personal hygiene in any household is at least 7.5–15 liters per person per day which means that those household who got the water and care for their drinking, cooking, and personal hygiene can prevent their children from diarrhea that can be transmitted by drinking water, cooking materials contamination, and by not keeping their personal hygiene safely. Water access of greater than 15 liters/day also reduces the chance of diarrhea occurrence by 93% which is incomparable with the result from ODF kebeles that indicate those households having 7.5–15 liters per person per day and households who access 15 liters per person per day which have no statistically significant association with under-five diarrhea. The result also showed that, in ODF kebeles, people who dispose solid wastes properly were 86% less likely to have diarrhea as compared to those who dispose improperly which is comparable with the result from OD kebeles in which safe disposal reduces the chance of diarrhea occurrence by 97%. This finding is in line with a study done in the Mecha district of West Gojjam [18]. This finding is explained by the fact that proper waste management reduces the risk of contamination and in turn risk of having diarrheal disease.

## 5. Strength and Limitation of the Study

The study explored factors that have an association with diarrhea in both ODF and OD kebeles comparatively against the standard guidelines. The study also used multiple methods of data collection like on-site observation to assess practice of latrine utilization. In addition, to assure the quality of the data, the standardized data collection tool was used and pretest was done before the actual data collection. As limitation, the study was cross-sectional which cannot measure the cause-and-effect relationship. There might also be a possibility of recall bias that will result in underreporting and misreporting of events. For more information, refer the thesis report [19].

## 6. Conclusion and Recommendations

The overall prevalence of under-five diarrhea among the individuals living in the ODF kebeles was lower as compared with the OD kebeles. Child immunization, latrine presence, water shortage in household, and solid waste disposal in ODF kebeles and per capita water consumption/water access at the individual level, water shortage in households, child immunization, and solid waste disposal in OD kebeles had statistically significant association with diarrhea occurrence in the Dangla district.

Ministry of health needs to reconsider the ODF status certification process of the kebeles which can be based on sustainability. Together with line ministries, regions, zones, woreda health structures, and stakeholders, the ministry has to improve child immunization, latrine presence and utilization, water shortage in household, and solid waste disposal practices that are directly linked with under-five diarrhea. Health workers and local authorities must give health education and sensitization for the community to improve OD practices. In addition, the findings of this study will serve as baseline evidence and pave the way for other researchers and policymakers to conduct more rigorous studies on this arena.

## Figures and Tables

**Table 1:** Participants characteristics in selected both ODF and OD kebeles in Dangla woreda, Northwest Ethiopia, 2016.

Variables		ODF		OD	
		Number	%	Number	%
Age (years)	15–24	31	11.8	17	6.5
	25–34	75	28.6	64	24.3
	35+	156	59.5	182	69.2

Sex	Female	162	61.8	112	42.6
	Male	100	38.2	151	57.4

Family size (mean = 5.53)	≤5	133	50.8	95	36.1
	>5	129	49.2	168	63.9

Marital status	Unmarried	8	3.1	5	1.9
	Married	228	87.0	235	89.4
	Divorced	10	3.8	13	4.9
	Widowed	16	6.1	10	3.8

Educational status of mother	Literate	87	33.2	90	34.2
	Illiterate	175	66.8	173	65.8

Educational status of father	Literate	148	56.5	120	45.6
	Illiterate	114	43.5	143	54.4

Occupation of mother	Farmer	198	75.6	207	78.7
	Housewife	64	24.4	56	21.3

Occupation of father	Farmer	249	95.0	251	95.4
	Private	13	5.0	12	4.6

Educated child in household	No	78	29.8	58	22.1
	Yes	184	70.2	205	77.9

Educational level of child	Higher education	19	10.3	35	17.1
	Primary/secondary education	144	78.3	153	74.6
	Read/write	21	11.4	17	8.3

Child immunization (any type)	No	52	19.8	110	41.8
	Yes	210	80.2	153	58.2

Average monthly net income (in ETB)	<1000	177	67.6	202	76.8
	1000 to 2000	77	29.4	31	11.8
	2001 and above	8	3.1	30	11.4

**Table 2:** Diarrhea occurrences among ODF and OD kebeles in Dangla woreda, Northwest Ethiopia, 2016.

Variables		ODF		OD		Chi-square (*P* value)
		Number	%	Number	%	
Diarrhea occurrence	No	236	90.1	168	63.9	50.791 (0.001)
	Yes	26	9.9	95	36.1	

Diarrhea persists	≤5 days	19	73.1	69	72.6	0.002 (0.964)
	>5 days	7	26.9	26	26.3	

Perception on sources of diarrhea	Food	21	43.8	51	41.1	51.535 (0.001)
	Water	26	54.2	71	57.3	
	Utensils	1	2.1	2	1.6	

Diarrhea prevention mechanisms done for the child	Nothing done	5	1.9	18	6.9	29.714 (0.001)
	Special food for the child	113	43.1	161	61.2	
	Clean food for the child	207	79.0	190	72.2	
	Care for hygiene of the child	160	61.1	209	79.5	
	Care for hygiene of the family	182	69.5	192	73.0	

**Table 3:** Households latrine ownership, the type of latrine, years for latrines construction, and household latrine utilization practice in Dangla woreda, Northwest Ethiopia, 2016.

Variables		Kebele status				Chi-square (*P* value)
		ODF		Non-ODF		
		Number	%	Number	%	
Latrine presence	No	27	10.3	19	7.2	1.56 (0.212)
	Yes	235	89.7	244	92.8	

Latrine type	Shared	6	2.3	13	4.9	3.97 (0.137)
	Traditional pit	229	87.4	231	87.8	

Years for latrine construction	<1 year	9	3.8	13	5.3	0.79 (0.852)
	1–2 years	72	30.6	76	31.1	
	2–3 years	113	48.1	111	45.5	
	3+ years	41	17.4	44	18.0	

Defecate openly after the latrine construction	No	205	87.2	165	62.7	27.74 (0.001)
	Yes	30	12.8	79	30.0	

**Table 4:** Households solid and liquid waste management in Dangla woreda, Northwest Ethiopia, 2016.

Variables		Kebele status				Chi-square (*P* value)
		ODF		OD		
		Number	%	Number	%	
Liquid waste disposal	Improper	90	34.4	156	59.3	32.85 (0.001)
	Proper	172	65.6	107	40.7	

Solid waste disposal	Proper	242	92.4	146	55.5	92.43 (0.001)
	Improper	20	7.6	117	44.5	

**Table 5:** Bivariate and multivariate analysis of variables with under-five diarrhea among ODF and OD kebeles in Dangla woreda, Northwest Ethiopia, 2016.

Variables		ODF		OD	
		COR (95% CI)	AOR (95% CI)	COR (95% CI)	AOR (95% CI)
Monthly income (in birr)	<1000	Ref		Ref	
	1000 to 2000	1.3 (0.111, 15.088)		0.374 (0.147, 1.002)	
	2001 to 3000	18.6 (0.065, 5173)		0.778 (0.346, 1.750)	

Family size	≤5	Ref	Ref	Ref	Ref
	>5	3.109 (1.260, 7.674)	0.715 (0.115, 4.443)	2.774 (1.565, 4.917)	0.840 (0.233, 3.035)

Education of mother	Literate	Ref		Ref	Ref
	Illiterate	0.775 (0.336, 1.787)		0.542 (0.321, 0.917)	0.512 (0.129, 2.035)

Occupation of mother	Farmer	Ref		Ref	Ref
	Housewife	1.429 (0.589, 3.462)		0.461 (0.233, 0.910)	2.431 (0.422, 13.995)

Child vaccination	No	Ref	Ref	Ref	Ref
	Yes	0.018 (0.005, 0.065)	0.037 (0.006, 0.243)^*∗*^	0.051 (0.027, 0.098)	0.032 (0.008, 0.123)^*∗*^

Latrine presence	No	Ref	Ref	Ref	Ref
	Yes	0.013 (0.004, 0.040)	0.036 (0.006, 0.233)^*∗*^	0.177 (0.062, 0.510)	0.382 (0.050, 2.913)

Main source of water	Improved	Ref		Ref	Ref
	Unimproved	1.059 (0.461, 2.434)		4.150 (2.100, 8.201)	1.640 (0.360, 7.472)

Distance of water source	<=1000	Ref	Ref	Ref	Ref
	>1000	6.320 (2.253, 17.726)	3.845 (0.291, 50.895)	9.506 (2.656, 34.019)	1.988 (0.239, 16.556)

Per capita water consumption (liters/day)	<7.4	Ref	Ref	Ref	Ref
	7.5–15	0.091 (0.032, 0.264)	0.332 (0.046, 2.405)	0.088 (0.044, 0.177)	0.029 (0.006, 0.152)^*∗*^
	>15.1	0.292 (0.099, 0.866)	0.527 (0.060, 4.600)	0.126 (0.056, 0.285)	0.068 (0.010, 0.474)^*∗*^

Water shortage	No	Ref	Ref	Ref	Ref
	Yes	13.768 (5.232, 36.229)	8.756 (1.13, 67.831)^*∗*^	10.828 (5.824, 20.13)	18.478 (4.692, 72.76)^*∗*^

Water treatment	No	Ref	Ref	Ref	Ref
	Yes	2.833 (1.138, 7.056)	1.884 (0.268, 13.271	2.907 (1.605, 5.267)	0.858 (0.166, 4.438)

Soap/Ash use yesterday	No	Ref		Ref	Ref
	Yes	0.873 (0.333, 2.293)		0.655 (0.374, 1.149)	

Liquid waste disposal	Dispose openly	Ref		Ref	Ref
	Dispose safely	0.484 (0.214, 1.095)		0.171 (0.092, 0.318)	0.435 (0.108, 1.751)

Solid waste disposal	Dispose openly	Ref	Ref	Ref	Ref
	Dispose safely	0.016 (0.005, 0.052)	0.143 (0.020, 0.998)^*∗*^	0.066 (0.035, 0.124)	0.023 (0.005, 0.117)^*∗*^

^*∗*^Indicates statistically significantly associated variables.

## Abbreviations

CLTSH: Community-led total sanitation and hygiene
EDHS: Ethiopian Demographic and Health Survey
FMOH: Federal Ministry of Health
HEP: Health Extension Program
HEW: Health Extension Workers
HMIS: Health Management Information System
MEDHS: Mini Ethiopian Demographic and Health Survey
MDG: Millennium Development Goals
OD: Open defecation
ODF: Open defection free
WASH: Water, Sanitation, and Hygiene
WHO: World Health Organization.
